# H89 enhances the sensitivity of cancer cells to glyceryl trinitrate through a purinergic receptor-dependent pathway

**DOI:** 10.18632/oncotarget.3124

**Published:** 2015-02-04

**Authors:** Marion Cortier, Rahamata Boina-Ali, Cindy Racoeur, Catherine Paul, Eric Solary, Jean-François Jeannin, Ali Bettaieb

**Affiliations:** ^1^ EPHE, Tumor Immunology and Immunotherapy Laboratory, Dijon, F-21000, France; ^2^ Inserm U866, Dijon, F-21000, France; ^3^ EA7269, University of Burgundy, Dijon, F-21000, France; ^4^ Inserm UMR1009, Gustave Roussy Institute, Villejuif F-94805, France; ^5^ University Paris-Sud, Faculty of Medicine, Le Kremlin-Bicêtre, F-94800, France

**Keywords:** H89, GTN, cancer, purinergic receptors, cGMP

## Abstract

High doses of the organic nitrate glyceryl trinitrate (GTN), a nitric oxide (NO) donor, are known to trigger apoptosis in human cancer cells. Here, we show that such a cytotoxic effect can be obtained with subtoxic concentrations of GTN when combined with H89, N-[2-(*p*-bromocinnamylamino)ethyl]-5-isoquinolinesulphonamide.2HCl. This synergistic effect requires the generation of reactive oxygen species (ROS) from H89 and NO from GTN treatment that causes cGMP production and PKG activation. Furthermore, the GTN/H89 synergy was attenuated by inhibition of P2-purinergic receptors with suramin and competition with ATP/UDP. By down-regulating genes with antisense oligonucleotides, P2-purinergic receptors P2X3, P2Y1, and P2Y6 were found to have a role in creating this cytotoxic effect. Thus, H89 likely acts as an ATP mimetic synergizing with GTN to trigger apoptosis in aggressive cancer cells.

## INTRODUCTION

In the last ten years, glyceryl trinitrate (GTN) has emerged as a potential new approach in cancer therapy. GTN is an organic nitrate compound [1] that is largely used to treat angina pectoris, congestive heart failure and acute myocardial infarction via the release of nitric oxide (NO) or related species [2]. In a phase II clinical trial, GTN improved the response rate to chemotherapy and increased the time to progression in patients with inoperable non-small-cell lung cancer [3]. Another phase II trial suggested that low doses of GTN could prevent prostate cancer progression after primary treatment failure [4]. Several ongoing clinical trials further explore the potential interest of using GTN in combination with other therapies in various cancer types (clinicaltrial.gov).

In preclinical studies, low concentrations of GTN (in the nanomolar range) were shown to prevent hypoxia-induced tumor cell evasion from immune cell surveillance [5] and to revert the chemoresistance of various types of cancer, including breast, melanoma and prostate cancers [6, 7]. We previously showed that higher concentrations of GTN (in the micromolar range) were required to induce caspase-mediated apoptosis of colon cancer cells and to sensitize these cells to Fas/CD95 ligand-mediated cell death [8]. Compared to other NO releasing compounds, GTN has a specific mode of action, i.e. it produces NO through biotransformation to nitrite by mitochondrial aldehyde dehydrogenase 2 (mtALDH) [9]. In colon cancer cells, NO-mediated apoptosis involves the activation of soluble guanylyl cyclase (sGC) and the production of cyclic GMP (cGMP), a potent protein kinase G (PKG) activator [10].

In the present study, we searched a strategy to decrease the dose at which GTN promotes apoptosis of cancer cells. We identified isoquinolinesulfonamide H89 (N-[2-(*p*-bromocinnamylamino) ethyl]-5-isoquinoline sulfonamide.2HCl) as a drug that synergizes with non-toxic concentrations of GTN. Surprisingly, this sensitizing effect of H89, which is mainly known as a protein kinase inhibitor, does not depend on kinase inhibition. We show that H89 interacts with P2 receptors and induces the production of reactive oxygen species that, combined with the NO generated by GTN, further activate the cGMP/PKG pathway and induce caspase-dependent cell death.

## RESULTS

### GTN synergizes with the kinase inhibitor H89 to induce apoptosis in colon cancer cells

**Figure 1:**
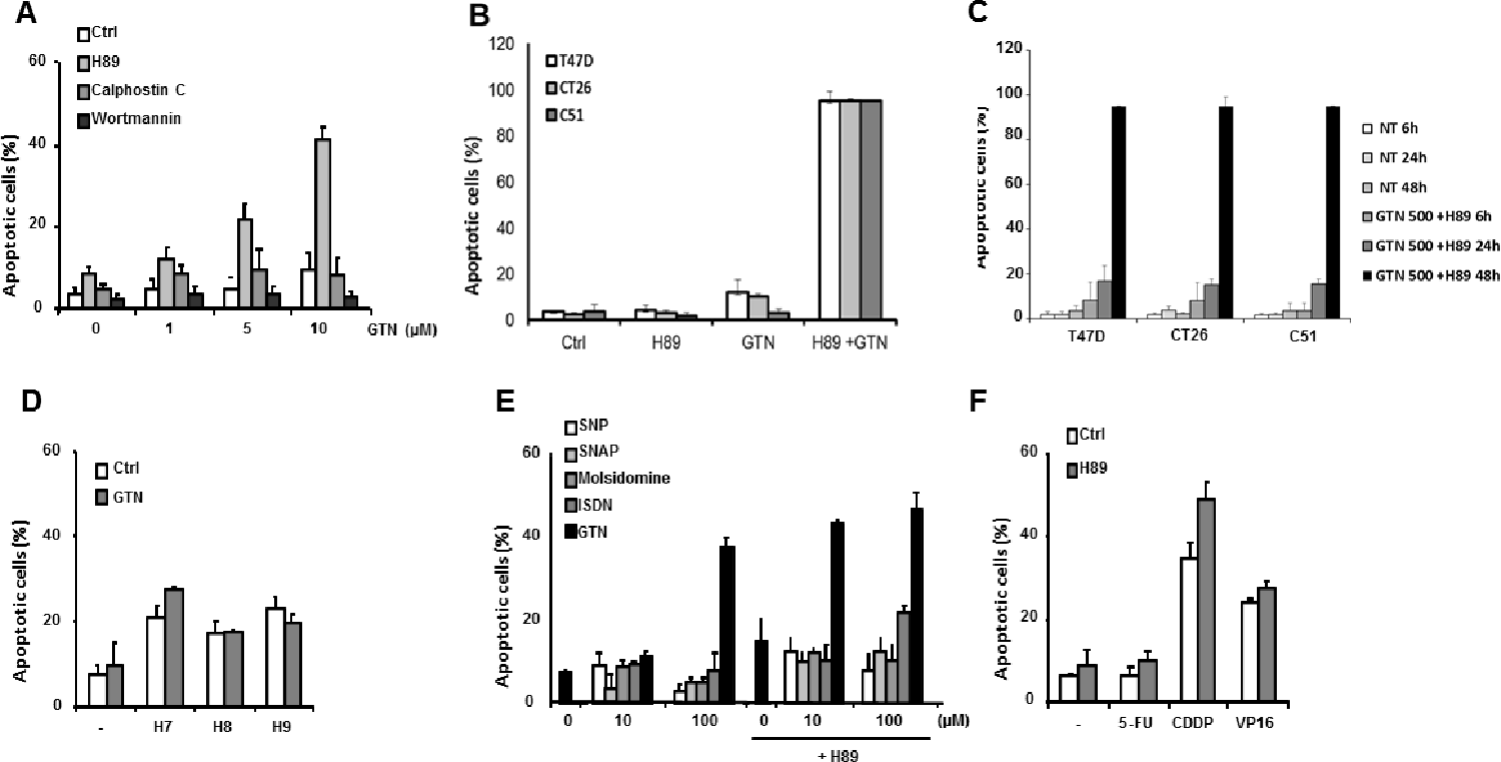
Characterization of SW480 cell sensitization to apoptosis by H89 Exponentially growing SW480 cells (3 × 10^5^/mL) were treated for 48 h at 37°C: **(A)** with the indicated concentrations of GTN and/or 10 μM H89, 100 nM calphostin C or 100 nM wortmannin; **(B)** CT26, C51 and T47D cells were treated for 48 h at 37°C with 500 μM GTN and/or 10 μM H89; **(C)** or for 6, 24 and 48 h at 37°C with 500 μM GTN and/or 10 μM H89. **(D)** SW480 cells were treated with 100 μM H7, H8 or H9 with or without 10 μM GTN; **(E)** with the indicated concentrations of different NO donors and/or 10 μM H89; or **(F)** with chemotherapeutic agents, 5 μg/mL 5-FU, 5 μg/mL CDDP or 50 μM VP16 and/or 10 μM H89. Apoptotic cells were identified by fluorescence microscopy after staining nuclei with Hoechst 33342. Results are the means of 3 independent experiments.

To determine whether inhibition of a cellular signalling pathway could sensitize cancer cells to GTN, we initially selected inhibitors of phosphatidyl inositide-3-kinase (Pi3K), Protein kinase C (PKC), and Protein kinase A (PKA) and tested their ability to induce apoptosis in cell lines exposed to non-toxic concentrations of GTN. Whereas the Pi3K inhibitor wortmannin, and the PKC inhibitor calphostin C did not demonstrate any significant effect, H89, an isoquinoline sulfonamide commonly used as a selective inhibitor of PKA, synergized with GTN in inducing apoptosis in 40% of SW480 colon cancer cells. More specifically, a non cytotoxic concentration of GTN (10 μM) combined with 10 μM H89 for 48 h induced apoptosis in more than 40% of SW480 cells compared to less than 10% in the control, with GTN alone, with H89 alone or with other GTN/inhibitor combinations, respectively (Figure 1A). The synergistic effect of GTN and H89 on SW480 cells was also observed in 2 murine colon cancer cell lines (CT26 and C51) and in a human mammary cancer cell line, T47D (Figure 1B) which was time dependent (Figure 1C). In subsequent experiments, we explored the molecular mechanism of this synergistic effect in SW480 cells.

Protein kinase inhibitors structurally related to H89 (H7, H8 and H9) were tested at concentrations of up to 100 μM but they did not produce a similar synergy with GTN (Figure 1D). H89 did not sensitize SW480 cells to any other NO donor tested at concentrations up to 100 μM, including sodium nitroprusside (SNP), S-nitroso-N-acetylpenicillamine (SNAP) and molsidomine exept isosorbide dinitrate (ISDN) which, at high concentration (100 μM) exhibited a modest synergistic effect when it was combined with H89 (Figure 1E). Also, H89 did not sensitize SW480 cells to chemotherapeutic drugs including 5-fluoro-uracil (5-FU, 5 μg/mL), cisplatin (CDDP, 5 μg/mL) and etoposide (VP16, 50 μM) (Figure 1F). Altogether, H89 appears to specifically synergize with GTN.

### The GTN/H89 combination induces caspase-dependent apoptosis

**Figure 2:**
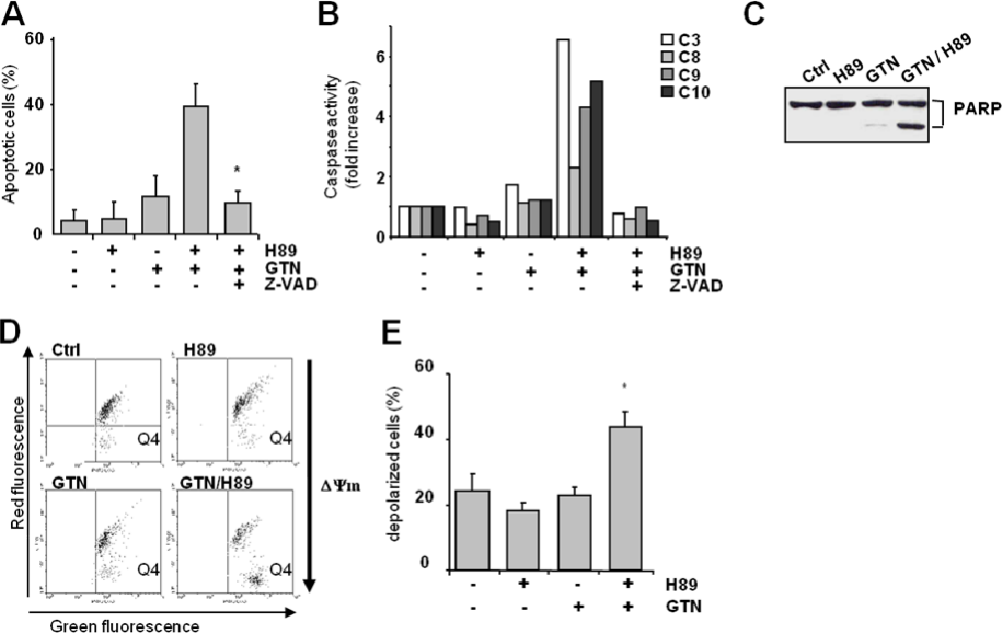
Characterization of GTN/H89-induced colon cancer cell apoptosis SW480 cells (3 × 10^5^/mL) were pretreated for 1 h with pan-caspase inhibitor z-VAD (75 μM) before exposure to GTN and/or H89 (10 μM each) for 48 h. **(A)** Apoptotic cells were counted after Hoechst 33342 staining. Results are the means of 3 independent experiments with SD shown as bars. *P < .05. **(B)** The cleavage of fluorigenic-specific peptides DEVD-AMC, IETD-AMC, LEHD-AFC and AEVD-AFC, respectively substrates of caspases-3 (C3), -8 (C8), -9 (C9) and -10 (C10), was measured in cell lysates. Data are from 1 experiment representative of 3 independent experiments. **(C)** Immunoblot analysis of PARP expression in SW480 cells exposed to 10 μM GTN and 10 μM H89 for 48 h. Data are from 1 experiment representative of 3 independent experiments. **(D)** SW480 cells were treated with GTN and H89 (10 μM each) for 48 h, then stained with 5 μM JC-1 probe before measuring cell fluorescence by flow cytometry, thus measuring ΔΨm. The dye fluoresces red when it aggregates in healthy mitochondria with a high membrane potential, whereas it fluoresces green in its monomeric form in mitochondria with a low membrane potential. The number of dots in the Q4 area (green fluorescence) indicates the number of cells with low ΔΨm. Results are from 1 experiment representative of 3 independent experiments. **(E)** Histogram showing the percentage of cells with a low ΔΨm, corresponding to the percentage of cells with fluorescence detected in the Q4 area. Results are the means of 3 independent experiments with SD shown as bars. *p < 0.05.

As high doses of GTN induce apoptosis in a caspase-dependent manner [11], the role of caspases in apoptosis induced by the combination of H89 with lower doses of GTN was investigated. Cell death induced by the H89/GTN combination was inhibited by a 1-h pre-treatment with the inhibitor zVAD-fmk (75 μM) (Figure 2A). By adding fluorogenic substrates to cell lysates we found that the drug combination activated enzymes that cleaved Ac-DEVD-AMC, Ac-LEHD-AMC and Ac-AEVD-AFC, suggesting caspase-3, caspase-9, and caspase-10 activation, and to a lesser extent, Ac-IETD-AMC, suggesting caspase-8 activation (Figure 2B). In addition, immunoblot analysis showed poly(ADP-ribose) polymerase (PARP), a well known caspase substrate, was cleaved in cells exposed to the H89/GTN combination (Figure 2C) and flow cytometry showed a dissipation of the mitochondrial transmembrane potential (ΔΨ m), an early requirement for apoptosis, as attested by the increase of the percentage of depolarized cells (Figure 2D, 2E).

### The sensitizing effect of H89 with GTN may not depend on kinase inhibition

**Figure 3:**
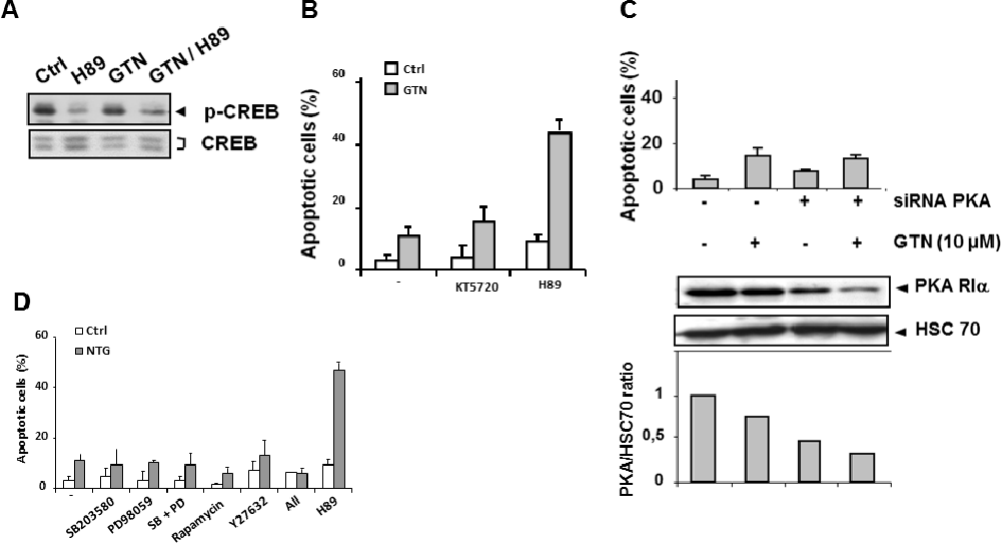
PKA is not involved in GTN/H89-induced apoptosis **(A)** Immunoblot analysis of CREB and its phosphorylated form in SW480 cells exposed to 10 μM GTN and 10 μM H89 for 48 h. The immunoblot shown is representative of 3 independent experiments. **(B)** Exponentially growing SW480 cells (3 × 10^5^/mL) were treated with 10 μM GTN and/or 10 μM H89 or 1 μM KT5720 for 48 h at 37°C. Apoptotic cells were counted after Hoechst 33342 staining. **(C)** Cells were transfected with a PKA-specific siRNA (20 μM) for 6 h then treated with 10 μM GTN for 48 h at 37°C. Then apoptotic cells were counted. Results are the means of 3 independent experiments. Total protein was isolated and analyzed by immunoblot for expression of PKA-RIα, the active subunit of PKA, quantified relative to HSC70 expression. One immunoblot representative of 3 independent experiments is shown. **(D)** SW480 cells were treated with the following protein kinase inhibitors 1 h before exposure to 10 μM GTN: 20 μM SB203580, 15 μM PD98059, 1 nM rapamycin, 10 μM Y27632, and 10 μM H89. ‘All’ refers to treatment with all inhibitors except H89. Apoptotic cells were counted. Results are the means of 3 independent experiments.

H89 was described as a potent inhibitor of PKA [12, 13]. H89 also inhibits p70 ribosomal S6 kinase-1 (S6K1), mitogen and stress-activated protein kinase-1 (MSK-1) and the Rho kinase ROCKII [14, 15]. SW480 cells were exposed to 10 μM H89, either alone or combined with 10 μM GTN, and this significantly inhibited phosphorylation of the transcription factor CREB, a downstream target of PKA [16] (Figure 3A). However, neither another selective inhibitor of PKA, KT5720 [17] (Figure 3B), nor the specific down-regulation of PKA regulatory chain alpha (PKA RIα) by RNA interference (Figure 3C), created an apoptosis-inducing synergy with GTN in SW480 cells. Similarly, S6K1 inhibition with rapamycin, MSK1 inhibition with SB203580 and PD98059 combination, and ROCK II inhibition with Y27632 did not mimic the sensitization of SW480 cells to GTN-induced apoptosis (Figure 3D).

### NO and cGMP signalling pathway are required for GTN/H89-mediated apoptosis

**Figure 4:**
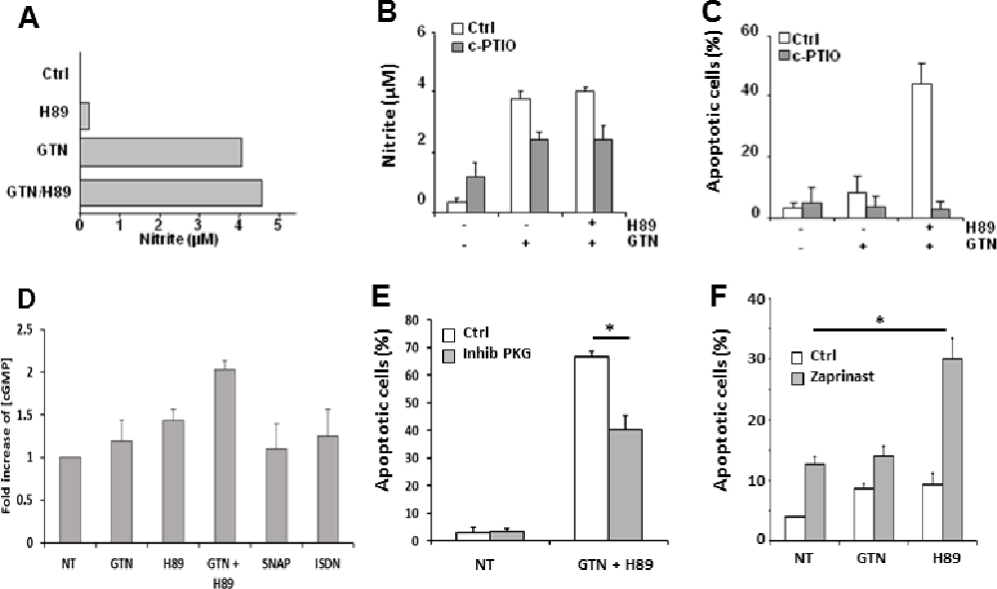
NO is involved in GTN/H89-induced apoptosis **(A)** Exponentially growing SW480 cells (3 × 10^5^/mL) were treated with 10 μM GTN and/or 10 μM H89 for 48 h at 37°C. The concentration of nitrite in the medium was quantified with the Griess method. **(B)** Exponentially growing SW480 cells (3 × 10^5^/mL) were treated with NO scavenger carboxy-PTIO (200 μM) for 1 h before exposure to 10 μM GTN and 10 μM H89 for 48 h at 37°C. The nitrite concentration in the medium was then measured and **(C)** apoptotic cells counted after Hoechst 33342 staining. Results are the means of 3 independent experiments. **(D)** cGMP content analysis using the colorimetric cGMP Direct immunoassay kit using lysates from SW480 cells treated with 10 μM GTN, 500 μM ISDN, 500 μM SNAP and/or 10 μM H89 for 16 h at 37°C. Data are from 1 experiment made in triplicate representative of three independent experiments **(E)** SW480 cells were treated with 500 μM Rp-8-Br-PET-cGMPS, a competitive inhibitor of PKG (inhib PKG), 10 μM GTN and 10 μM H89 for 48 h at 37°C, and apoptotic cells were counted. Results are the means of 3 independent experiments. *P < .05. **(F)** SW480 cells were treated with 200 μM zaprinast a phosphodiesterase 5 inhibitor, 10 μM GTN and 10 μM H89 for 48 h at 37°C. Results are the means of 3 independent experiments. *P < .05.

As a NO donor, the contribution of GTN to the synergistic effect of the GTN/H89 combination is likely due to the release of NO. Exposure of SW480 cells to 10 μM GTN induces the release of nitrite in the culture medium (> 4 μM at 48 h compared to < 1 μM in controls; Figure 4A). H89 did not significantly increase the GTN-induced NO production (Figure 4A). Interestingly, the NO scavenger (4-carboxyphenyl)-4,4,5,5-tetramethylimidazoline-1-oxyl-3-oxide (c-PTIO) significantly lowered GTN and GTN/H89-induced nitrite levels (Figure 4B), and abrogated GTN/H89-mediated apoptosis (Figure 4C). This suggests that NO production was required for the combination to induce apoptosis.

Since NO is a potent activator of guanylyl cyclase/cGMP/PKG signalling pathway [18], we investigated the possible involvement of this pathway in the GTN/H89 combination induced-apoptosis. Exposure of SW480 cells to 10 μM GTN, 10 μM H89 or H89/GTN combination synergistically increased the intracellular level of cGMP as evaluated by ELISA (Figure 4D). Of note, the inability of other NO donors to synergize with H89 was not due to their inability to induce cGMP production, since SNAP and ISDN induced cGMP production (Figure 4D). This signalling intermediate seems to be involved in H89/GTN-mediated cell apoptosis since exposure of SW480 cells to 500 μM Rp-8-Br-PET-cGMPS, a competitive inhibitor of cGMP-dependent protein kinase (PKG), significantly decreased the sensitization of SW480 cells to the combination (Figure 4E). Furthermore, the inhibition of phosphodiesterase by zaprinast, known to increase cGMP level, rendered cells more sensitive to H89-induced cell apoptosis (Figure 4F), indicating that upregulation of cGMP and then PKG activation were required for the combination to induce apoptosis.

### ROS production is involved in GTN/H89-mediated apoptosis

**Figure 5:**
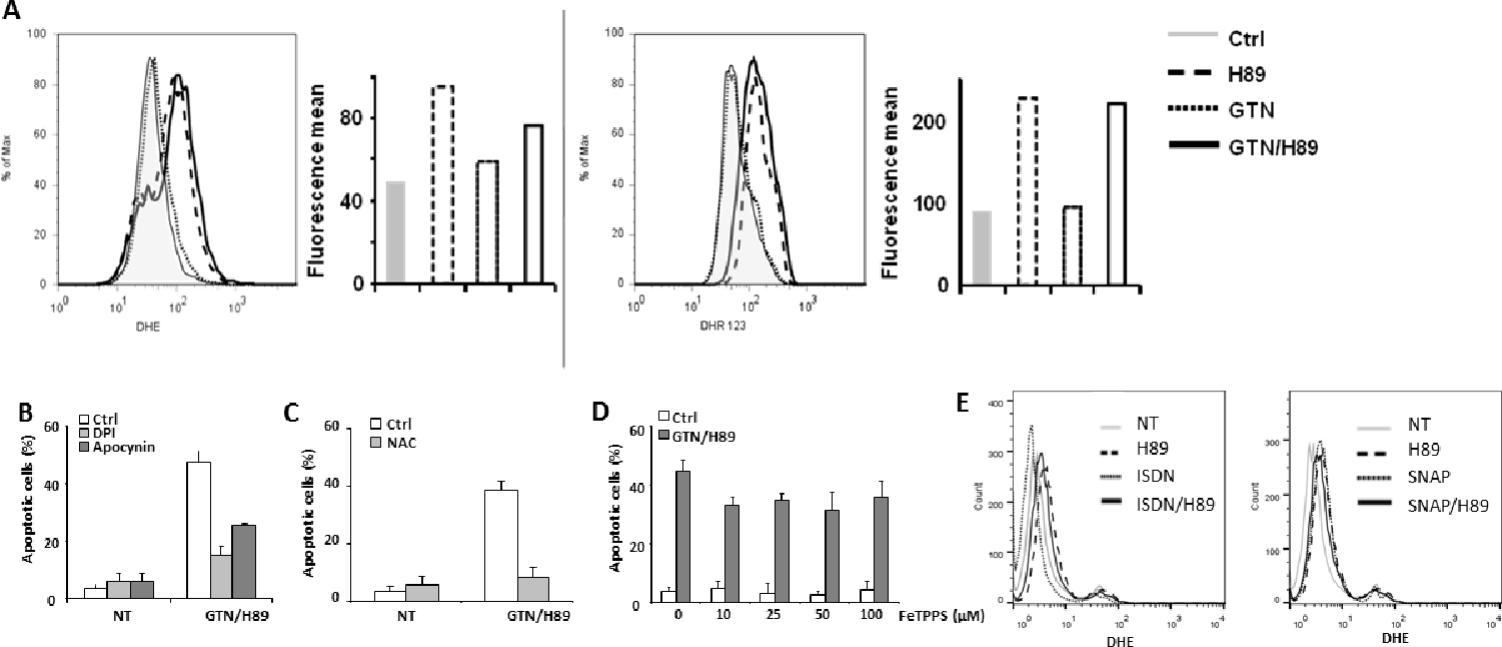
ROS is involved in GTN/H89-induced apoptosis **(A)** SW480 cells were treated with 10 μM GTN and 10 μM H89 for 48 h at 37°C. Then the amounts of ROS in cells were monitored. Left, the relative amount of superoxide anion per cell was monitored by flow cytometry using the DHE dye. Right, the relative amount of hydrogen peroxide per cell was monitored by flow cytometry using the DHR123 dye. One readout representative of six experiments is shown for each dye. The horizontal axis shows the geometric green fluorescence intensity and the vertical axis shows the percentage of cells. Before exposure to 10 μM GTN and 10 μM H89 for 48 h at 37°C, exponentially growing SW480 cells (3 × 10^5^/mL) were treated for 1 h **(B)** with ROS production inhibitors, DPI (5 μM) and apocynin (500 μM), **(C)** with 10 mM N-acetylcysteine (NAC), or **(D)** with ONOO^−^ scavenger FeTPPS at the indicated concentrations. Apoptotic cells were counted after Hoechst 33342 staining. Results are the means of 3 independent experiments. **(E)** SW480 cells were treated with 500 μM ISDN, 500 μM SNAP and/or 10 μM H89 for 48 h at 37°C. The relative amount of superoxide anion per cell was monitored by flow cytometry using the DHE dye. Results are representative of 2 independent experiments.

As NO utilization is obligatory linked to the mitochondrial production of reactive oxygen species (ROS) [19], the possible involvement of ROS in apoptosis induced by the GTN/H89 combination was investigated. Exposure of SW480 cells to 10 μM H89 for 48 h induced the production of ROS including superoxide anions (O_2_^−^) and H_2_O_2_, as evaluated by flow cytometry using cell-permeable dihydroethamine (DHE) and dihydrorhodamine 123 (DHR123), respectively (Figure 5A). GTN alone, at 10 μM for 48 h, did not induce the production of ROS or did not increase ROS production when combined with H89 (Figure 5A). In order to determine the origin of ROS produced in H89-treated cells, we investigated the involvement of nicotinamide adenine dinucleotide phosphate (NADPH) oxidase, a well known enzyme that catalyzes ROS production. The combination's ability to trigger apoptosis was attenuated by approximately 60 and 40% by two NADPH oxidase inhibitors [20], namely diphenylene iodonium (DPI) and apocynin, respectively (Figure 5B), suggesting that this enzyme is required for the superoxide production resulting from exposure to H89. ROS production may be required for the H89/GTN combination to induce apoptosis as the latter was prevented by the antioxidant N-acetyl-L-cysteine (NAC) (Figure 5C). Because NO and superoxide anions together generate peroxynitrite, a high toxic compound, we wondered whether this oxidative agent was responsible for GTN/H89-induced apoptosis. Treatment of cells with the peroxynitrite scavenger FeTPPS [21] (at concentrations up to 100 μM) did not significantly affect the sensitivity of SW480 cells to the H89/GTN combination (Figure 5D), suggesting that peroxynitrite generation was not involved in apoptosis induction. Of note, one can speculate that the failure of the other NO donors to synergize with H89 could be due to their capacity to inhibit H89 activity. To address this question, we evaluated their impact on H89-induced ROS production. Exposure of SW480 cells to SNAP or ISDN and H89 did not abolish the ability of H89 to induce ROS production (Figure 5E).

### GTN/H89-induced apoptosis involves ATP receptors

**Figure 6:**
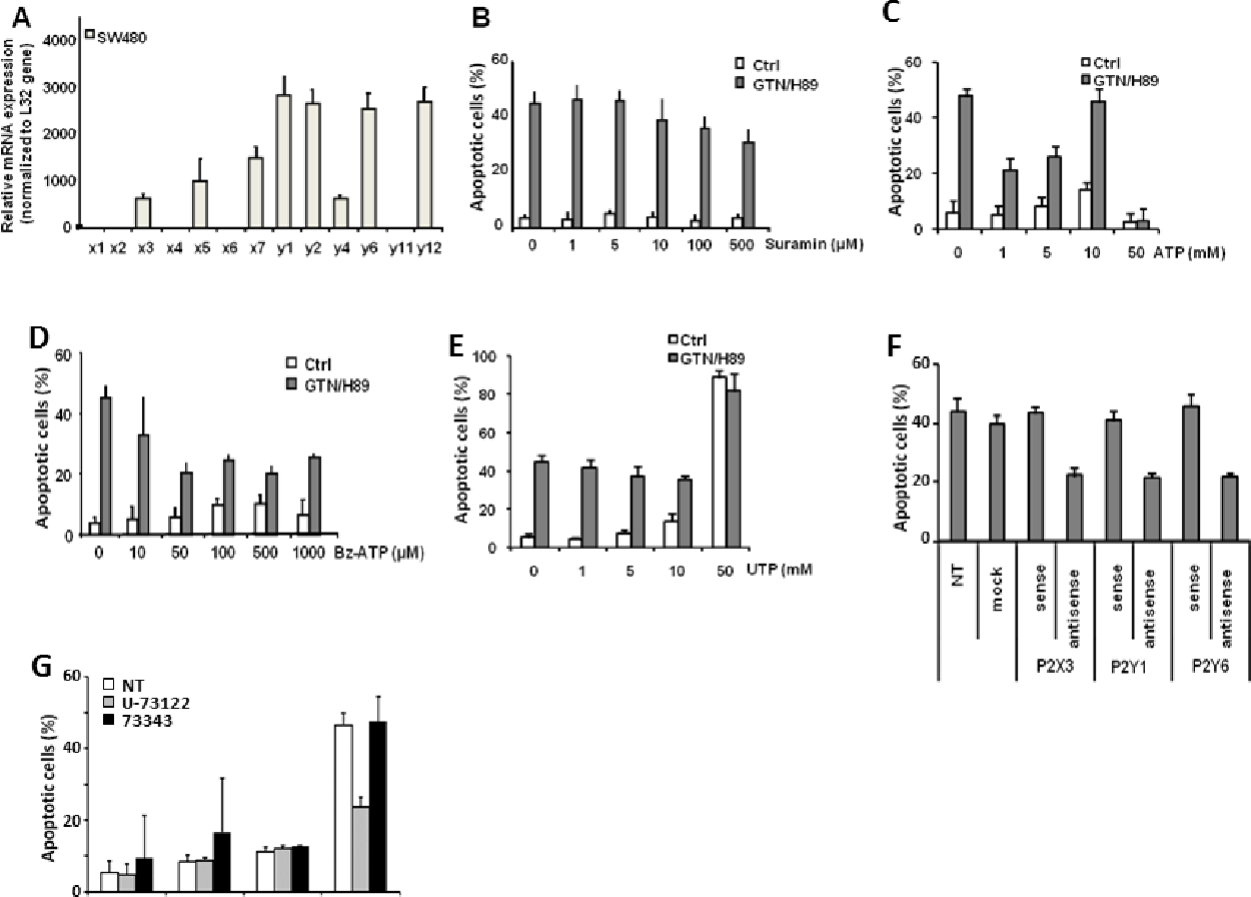
Purinergic receptors participate in GTN/H89-induced apoptosis **(A)** RQ-PCR analysis of expression of the indicated genes in SW480 cells, normalized to L32 gene expression. Exponentially growing SW480 cells (3 × 10^5^/mL) were treated with 10 μM GTN or/and 10 μM H89 for 48 h at 37°C with suramin **(B)**, ATP **(C)**, Bz-ATP **(D)** and UTP **(E)** at the indicated concentrations. Apoptotic cells were counted after Hoechst 33342 staining. **(F)** SW480 cells were transfected with P2X3, P2Y1 and P2Y6-targeted sense or antisense oligonucleotides, 24 h before treatment with 10 μM GTN and 10 μM H89 for 48 h at 37°C. Non transfected cells (NT) and cells transfected without oligonucleotides (mock) were used as controls. Apoptotic cells were counted after Hoechst 33342 staining. **(G)** SW480 cells were treated with 10 μM GTN and 10 μM H89 in the presence of 2.5 μM U-73122, a phospholipase C inhibitor, or 2.5 μM of its inactive analog U-73343. Apoptotic cells were counted after Hoechst 33342 staining. Results are the means of 3 independent experiments. *P < .05.

H89 was suggested to competitively inhibit enzyme activities at the ATP-binding site of various kinases [22]. We wondered whether this ability to compete with ATP could be extended to purinergic receptors and could account for its synergistic effect with GTN. RQ-PCR was used to test the expression of different purinergic receptors in SW480 cells: P2X(3, 5, 7)R, and P2Y(1, 2, 4, 6, 12)R are expressed whereas P2X(1, 2, 4, 6)R and P2Y(11)R mRNAs were not detected (Figure 6A). Exposure of SW480 cells to suramin, a nonselective inhibitor of purinergic receptors, induced a slight, dose-dependent decrease in GTN/H89-induced apoptosis (Figure 6B). Agonists of purinergic receptors such as ATP (Figure 6C) and 3′-O-(4-benzoyl)benzoyl-ATP (Bz-ATP), a more potent prototypic P2X7 receptor agonist (Figure 6D) dose-dependently impaired induction of apoptosis by the H89/GTN combination whereas UTP, another agonist of purinergic receptors, was ineffective (Figure 6E). Of note, several other ligands for purinergic receptors such as ATP, UDP, meATP, and TNP-ATP were ineffective to synergize with GTN to induced apoptosis (data not shown). Cells were transfected with antisense oligonucleotides specific to P2X3, P2Y1 and P2Y6, down-regulating the expression of these receptors. In these cells H89/GTN-induced apoptosis was prevented (Figure 6F) further suggesting a role for these receptors in cell death induction. As phospholipase C beta (PLCβ) is a downstream effector of purinergic receptor signaling, the possible involvement of this enzyme in H89 effect was investigated. Treatment of SW480 cells with a PLC-selective inhibitor U-73122 (at 2.5 μM) reduced apoptosis induced by H89/GTN combination, whereas the inactive analog of U-73122, U-73343, did not (Figure 6G), suggesting that activation of PLC-β was required for the combination to induce apoptosis.

## DISCUSSION

High doses of GTN can induce cancer cell apoptosis [23, 24, 8] and this compound could be efficient as an adjuvant to chemotherapy in the treatment of human cancers [3]. The present study suggests that the efficacy of GTN could be improved by combining it with the isoquinolinesulfonamide H89, commonly described as a selective inhibitor of protein kinases, especially protein kinase A. Surprisingly enough, the efficacy of the combination does not depend on the kinase inhibition properties of the drug. The H89/GTN combination triggers a caspase-dependent apoptosis that involves a cGMP-dependent mechanism through NO production induced by GTN and ROS production induced by H89. This latter conclusion is supported by previous reports in which increase of cellular cGMP and activation of PKG induced apoptosis in SW480 cells [25].

The effects of H89 appear to require several P2 receptors. P2-purinergic receptors can modulate proliferation, apoptosis [26, 27] and differentiation [28] in malignant cells. Identified targets of H89 include P2X3, which is one of the seven ionotropic non-selective monovalent gating cation channels, and P2Y1 and P2Y6, which are two of the eight members of the P2-purinergic receptor family that are coupled to trimeric G proteins [29]. Activation of P2X7 and P2Y1 receptors with high concentrations of extracellular ATP triggered colon cancer cell apoptosis [26], whereas low concentrations of ATP or UTP stimulated cell proliferation through P2Y1 and a downstream pathway that involves PKC, Src kinases and cell surface metalloproteases [26, 27]. Activation of the P2Y6 receptor has also been reported to protect normal cells from apoptosis induced by TNF-α [30–32]. We show here that exposure of cells to purinergic receptor agonists such as ATP clearly affects the GTN and H89 synergy. Although we have not demonstrated how the inhibition of purinergic receptors affects the synergistic activity of H89, one can speculate, however, that these receptor inhibitors could prevent H89 binding to the corresponding purinergic receptors.

The H89 molecule is thought to compete with ATP at its binding site within some protein kinases [22]. It may also compete with ATP in binding to some purinergic receptors. In the cell line studied, H89 appears to inhibit an anti-apoptotic signal mediated by these receptors, thus sensitizing cancer cells to GTN-induced apoptosis. The effect is very specific as it could not be reproduced by other molecules of the isoquinolinesulfonamide family, namely H7, H8, and H9. In addition, although NO release from GTN is required for the observed apoptosis, combining H89 with other NO donors does not reproduce the synergy of the H89/GTN combination. The discrepancy between GTN and the other NO donors to synergize with H89 was not due to a possible inhibition of H89 activity by the other NO donors, since neither isosorbide dinitrate nor SNAP were able to affect H89-mediated ROS production, but may be related to the intracellular location of NO, therefore affecting different targets. Indeed, whether almost NO donors release spontaneously NO in aqueous medium and reach extracellular and intracellular proteins, GTN undergoes biotransformation by mtALDH, which occurs in mitochondria [33], leading to local NO accumulation and modification of mitochondrial proteins. Mitochondrial proteins that can be modified by nitrosylation include the F1-ATPase alpha1 subunit [34], complex II (succinate: ubiquinone oxidoreductase) [35], and complex III (ubiquinol–cytochrome c reductase) [36]. S-nitrosylation of cytochrome c oxidase causes the mitochondrial membrane potential to dissipate [37]. Complex I S-nitrosylation is a factor in Parkinson disease [38].

Our study also shows that cGMP/PKG signaling plays a key role in GTN and H89 synergy. Indeed, the increasing of cGMP induced by GTN and H89 (Figure 4D) and presumably by an inhibitor of PDE5 (a catabolism enzyme of cGMP) dictates the synergistic effect between GTN an H89. Although, we are not able to definitely demonstrate that the cGMP signaling alone is sufficient to induce this synergy, our results favor this hypothesis. Indeed, as shown in Figure 4E, the PDE5 inhibitor, known to stabilize cGMP, induced apoptosis in cancer cells. ROS generation could possibly occur upstream of cGMP signaling in GTN/H89-mediated apoptosis [39].

Mitochondrion is also a major source of oxygen free radicals in the form of superoxide [40, 41]. It is unlikely to be the site of production of this oxygen radical in cells exposed to H89 since inhibition of the different mitochondrial complexes involved in superoxide generation did not mimic H89 cooperation with GTN in inducing cancer cell apoptosis (not shown). By contrast pharmacological inhibition of another major source of superoxide, the glycolytic NADPH oxidase enzyme, mainly present in the cytosol [42], significantly reduced GTN/H89-mediated cell death. These results highlight the fact that the cooperation between NO and superoxide in inducing cell death is somehow dependent on their localized subcellular production.

To conclude, we suggest that the promising results obtained with GTN in treating malignant tumors could be improved by combining it with H89 to eliminate cancer cells that express P2 receptors through inducing their death by apoptosis.

## METHODS

### Drugs and reagents

GTN was purchased from Merck (Lyon, France). H89 [N-[2-(*p*-bromocinnamylamino)ethyl]-5-isoquinolinesulfonamide.2HCl], H7, H8, and H9 were purchased from Tebu-bio (Le Perray en Yvelines, France). H89 (like H7, H8 and H9) is a member of the isoquinolinesulfonamide group of protein kinase inhibitors and tends to exhibit selective inhibition of protein kinase A (PKA) [12, 43]. Rp-8-bromo-b-phenyl-1,N2-ethenoguanosine 3′:5′-cyclic monophosphorothionate sodium (Rp-8-Br-PET-cGMPS), a competitive inhibitor of cGMP-dependent protein kinase G (PKG) was from Sigma-Aldrich (Saint Quentin Fallavier, France). The broad spectrum caspase inhibitor Z benzyloxycarbonyl-Val-Ala-Asp-fluoromethylketone (Z-VAD-fmk) was purchased from Bachem (Weil am Rhein, Germany), KT5720 and FeTPPS from VWR (Strasbourg, France), a colorimetric cGMP Direct immunoassay kit from Biovision (Milpitas, CA) and all other chemicals and reagents from Sigma-Aldrich or local suppliers.

### Cell culture and transfection

The human SW480 and the murine CT26 and C51 colorectal cancer cells and the human T47D mammary cancer cells were purchased from American Tissue Culture Collection (Manassas, VA). Cells were grown in a 1:1 (vol/vol) mixture of DMEM and HAM-F10 (Biowhittaker, Fontenay-sous-Bois, France) supplemented with 5% fetal calf serum (FCS) (Gibco BRL, Eriny, France) and 2 mmol/L L-glutamine at 37°C in a dry atmosphere. Cells were routinely detached with 0.125% trypsin, 0.1% ethylenediaminetetraacetic acid (EDTA) and washed once in the culture medium before treatment. Transfection was done by adding 5 μg of plasmid DNA to 3 × 10^6^ cells for 4 h in the presence of Superfect transfection reagent (Qiagen, Courtaboeuf, France). Transfected cells were harvested 24 h later. Cell viability was not affected by the transfection. The PKA siRNA assay kit used for transfection (Upstate, Dundee, UK) contained 4 pooled selected siRNA duplexes each with “UU” overhangs and a 5′ phosphate on the antisense strand.

Antisense nucleotides (5 μM) were used to downregulate candidate purinergic receptors in SW480 cells as described [44] using the Superfect and the Lipofectamine 2000™ transfection reagents as outlined by the manufacturer (Invitrogen, Cergy Pontoise, France).

### Quantification of nitrates and nitrites

NO production was determined indirectly by measuring the amount of nitrite and nitrate in cell culture media using the Griess microassay as described [45]. Briefly, 100 μL of culture supernatant was added to 100 μL of Griess reagent (0.5% sulfanilamide and 0.05% naphtylethylenediamine in 2.5% phosphoric acid). Absorbance was measured at 540 nm in a multiwell microtiter plate reader. Nitrite concentrations were calculated by comparison with a standard sodium nitrite solution.

### Counting of apoptotic cells

Cells (3 × 10^5^ per mL of culture) were treated with 10 μM GTN and/or 10 μM H89 for 48 h at 37°C. After treatment, the whole population of cells (including plastic-attached and floating cells) was washed in cold phosphate buffer saline (PBS) and exposed for 15 min to 1 μg/mL Hoechst 33342 at 37°C. Fluorescence microscopy was used to view the differences in chromatin staining and the percentage of apoptotic cells (those showing chromatin condensation and nuclear fragmentation) was determined by scoring 300 cells for each sample.

### Caspase activity measurement

Cells were incubated in lysis buffer (150 mM NaCl, 50 mM Tris-HCl pH 8.0, 0.1% SDS, 1% Nonidet P-40 (NP-40), 0.5% sodium deoxycholate) for 30 min at 4°C and centrifuged at 10 000 *g* for 20 min at 4°C. Samples of the supernatant (50 μg protein) were incubated in assay buffer (100 mM HEPES (N-2-hydroxyethylpiperazine-N’-2-ethanesulfonic acid) pH 7.0,1 mM EDTA, 0.1% CHAPS (3-[(3-cholamidopropyl) dimethylammonio]-1-propane sulfate), 10% glycerol, 20 mM dithiothreitol) in the presence of 100 μM fluorogenic peptide substrates: Ac-DEVD-AMC, Ac-IETD-AMC, Ac-LEHD-AFC (Biomol, Plymouth Meeting, PA, USA) or Ac-AEVD-AFC (R&D Systems, Minneapolis, MN, USA). Released AMC and AFC were excited at 380 nm and 400 nm and emission measured at 460 and 505 nm, respectively. Fluorescence was monitored continuously at 37°C for 30 min in a dual luminescence fluorimeter (MicroTek OS, Bio-Tek Kontron Instruments, Winooski, VT, USA).

### Immunoblot analysis

Cells (3 × 10^5^ per mL of culture) were washed twice with cold PBS and boiled in buffer (1% sodium dodecyl sulfate (SDS), 1 mmol/L sodium-orthovanadate, 10 mmol/L Tris pH 7.4) in the presence of a cocktail of protease inhibitors (Roche) for 10 min at 4°C. The viscosity of the samples was reduced by ultrasound. Lysates were harvested and the protein concentration was measured using a Bio-Rad DC protein assay kit. Samples containing 50 μg of protein were incubated in loading buffer (125 mmol/L Tris-HCl pH 6.8, 10% β-mercaptoethanol, 4.6% SDS, 20% glycerol and 0.003% bromophenol blue), separated by sodium dodecyl sulfate polyacrylamide gel electrophoresis (SDS-PAGE) and blotted onto PVDF membrane (BioRad). After blocking non-specific binding sites for 2 h with 8% non fat milk in 0.1% Tween 20 in PBS (TPBS), the membrane was incubated overnight at 4°C with primary antibodies (Abs): anti-PARP (Cell Signaling, Saint Quentin Yvelines, France), anti-CREB (Euromedex, Mundolsheim, France), anti phospho-CREB (Cell Signaling), anti-PKA RIα (BD Transduction Laboratories, Le Pont de Claix, France) or anti-HSC-70 (Santa Cruz Biotechnology, Santa Cruz, CA). After three washes in TPBS, the membrane was incubated with horseradish peroxidase-conjugated goat anti-mouse or anti-rabbit Abs as appropriate for 30 min at room temperature, then washed three times in TPBS. An enhanced chemiluminescence detection kit (Luminol, Santa Cruz) and autoradiography were used to develop immunoblots.

### Detection of ΔΨm

The ΔΨm was measured by flow cytometry using the lipophilic cation JC-1 (5,5′, 6,6′-tetrachloro-1,1′,3,3′-tetraethylbenzimidazol-carbocyanine iodide) (Molecular Probes, Invitrogen, Germany). JC-1 stains mitochondria in cells with high mitochondrial membrane potentials by forming orange-red fluorescent J-aggregates that emit at 590 nm upon excitation at 490 nm. In cells with depolarized or damaged mitochondria, JC-1 becomes monomeric and emits at 525 nm with the 490 nm excitation wavelength. Cells were incubated with 5 μg/ml JC-1 for 30 min at 37°C in the dark. Cells were then washed in PBS and analyzed immediately in a flow cytometer (LSRII, BD Biosciences). A total of 10 000 cells were analyzed for green fluorescence with a 525-nm filter and for orange fluorescence with a 590-nm filter. All data were analyzed with FlowJo.

### Detection of intracellular reactive oxygen species (ROS)

Intracellular peroxide and superoxide levels in cells were assessed using dihydrorhodamine 123 (DHR123) and dihydroethidium (DHE) (Molecular Probe Inc., Eugene, OR, USA) using a flow cytometer (LSRII; BD Biosciences). DHE is mainly oxidized by superoxide anions while DHR123 reacts with superoxide anion and hydrogen peroxide. A total of 10 000 cells were analyzed within 60 min of staining.

### cGMP assay

SW480 cells (3 × 10^5^ per mL of culture) were treated with 10 μM GTN and/or 10 μM H89 for 16 h. Cells were then lysed and assayed for cGMP content using the colorimetric cGMP direct immunoassay kit (Biovision, Milpitas, CA). The assay was performed according to the manufacturer's specifications.

### Gene expression analysis

Total RNA was isolated with Trizol (Invitrogen) and reverse transcribed with Moloney virus reverse transcriptase (Promega, Madison, WI, USA) primed with random hexamers (Promega). Real-time quantitative PCR (RQ-PCR) was performed with AmpliTaq Gold polymerase in an Applied Biosystem 7500 Taq thermocycler using the standard SYBR Green detection protocol as outlined by the manufacturer (Applied Biosystems, Foster City, CA, USA). Briefly, 12 ng of cDNA, 50 nM (each) primers and 1 × SYBR Green mixture were used in a total reaction volume of 20 μl. Primer sequences will be given upon request.
